# The Effects of Epigallocatechin-3-Gallate Nutritional Supplementation in the Management of Multiple Sclerosis: A Systematic Review of Clinical Trials

**DOI:** 10.3390/nu16162723

**Published:** 2024-08-15

**Authors:** Amanda Claudia Schuldesz, Raluca Tudor, Prashant Sunil Nandarge, Ahmed Elagez, Amalia Cornea, Radu Ion, Felix Bratosin, Mihaela Prodan, Mihaela Simu

**Affiliations:** 1 Doctoral School, “Victor Babes” University of Medicine and Pharmacy Timisoara, 300041 Timisoara, Romania; amanda.schuldesz@umft.ro (A.C.S.); mihaela.prodan@umft.ro (M.P.); 2 Discipline of Neurology, “Victor Babes” University of Medicine and Pharmacy Timisoara, 300041 Timisoara, Romania; amalia.cornea@umft.ro (A.C.); simu.mihaela@umft.ro (M.S.); 3 Department of General Medicine, D.Y. Patil Medical College Kolhapur, Kolhapur 416005, India; prashantnandarge1997@gmail.com; 4 Department of General Medicine, Misr University for Science & Technology, Giza 3236101, Egypt; ahmeddmahmouudd@gmail.com; 5 Department III Functional Sciences, Division of Public Health and Management, Victor Babes University of Medicine and Pharmacy, 300041 Timisoara, Romania; radu.ion@umft.ro; 6 Methodological and Infectious Diseases Research Center, Department of Infectious Diseases, Victor Babes University of Medicine and Pharmacy, 300041 Timisoara, Romania; felix.bratosin@umft.ro

**Keywords:** systematic review, epigallocatechin gallate, multiple sclerosis, dietary supplements

## Abstract

Multiple sclerosis (MS) is a chronic, debilitating neurological condition for which current treatments often focus on managing symptoms without curing the underlying disease. Recent studies have suggested that dietary supplements could potentially modify disease progression and enhance quality of life. This systematic review aims to evaluate the efficacy and safety of epigallocatechin-3-gallate (EGCG) as a dietary supplement in patients with MS, with a specific focus on its impact on disease progression, symptom management, and overall quality of life. We conducted a comprehensive systematic review following Preferred Reporting Items for Systematic Reviews and Meta-Analyses (PRISMA) guidelines, utilizing an exhaustive search across the databases PubMed, Scopus, and Web of Science up to 23 February 2024. Eligible studies were randomized controlled trials. Nine clinical trials involving 318 participants were analyzed, with dosages ranging from 600 mg to 1200 mg of EGCG daily, although most studies had only a 4-month follow-up period. Results indicated that EGCG supplementation, particularly when combined with coconut oil, led to significant improvements in metabolic health markers and functional abilities such as gait speed and balance. One trial observed significant improvements in the Berg balance scale score from an average of 49 to 52 after four months of treatment with 800 mg of EGCG daily. Additionally, interleukin-6 levels significantly decreased, suggesting anti-inflammatory effects. Measures of quality of life such as the Beck Depression Inventory (BDI) scale showed significant improvements after EGCG supplementation. However, primary outcomes like disease progression measured by the Expanded Disability Status Scale (EDSS) and Magnetic Resonance Imaging (MRI) of lesion activities showed minimal or no significant changes across most studies. EGCG supplementation appears to provide certain symptomatic and functional benefits in MS patients, particularly in terms of metabolic health and physical functionality. However, it does not significantly impact the primary disease progression markers such as EDSS scores and MRI lesions. These findings underscore the potential of EGCG as a supportive treatment in MS management, though its role in altering disease progression remains unclear. Future research should focus on long-term effects and optimal dosing to further elucidate its therapeutic potential.

## 1. Introduction

Multiple sclerosis (MS) is a chronic, immune-mediated disease process characterized by inflammation, demyelination, and subsequent axonal damage within the central nervous system (CNS). It has high heterogeneity in its progression and symptoms, which complicates its management, necessitating a multifaceted therapeutic approach [1,2]. Although the precise origin of MS remains elusive, it is widely recognized as a multifactorial disease, influenced by environmental factors in conjunction with genetic susceptibility, the exact interplay of which is still not fully understood today.

Currently, the management of MS focuses on immunomodulatory therapies, symptomatic treatment, and lifestyle modifications to slow disease progression and alleviate symptoms [3,4,5]. While several disease-modifying therapies (DMTs) such as interferon-beta, glatiramer acetate, and natalizumab have shown efficacy in reducing relapses and slowing disease progression in multiple sclerosis, their side effects and long-term impacts raise concerns [6,7,8]. For instance, some of them can cause flu-like symptoms and liver damage, while others carry the risk of progressive multifocal leukoencephalopathy [9,10]. Moreover, long-term use of these DMTs involves risks of malignancy and persistent immunosuppression, complicating treatment decisions and underscoring the need for safer therapeutic alternatives [11].

Dietary supplementation has emerged as an adjunctive treatment strategy in MS, with various natural compounds being investigated for their potential to enhance immune regulation and neuroprotection [12], such as the potential supplementation of Vitamin D, Coenzyme Q10, melatonin, or probiotics, although with varying effects [13,14]. Among these, epigallocatechin-3-gallate (EGCG), a major polyphenolic compound found in green tea, has attracted significant interest due to its antioxidant and anti-inflammatory properties [15].

**Figure 1:**
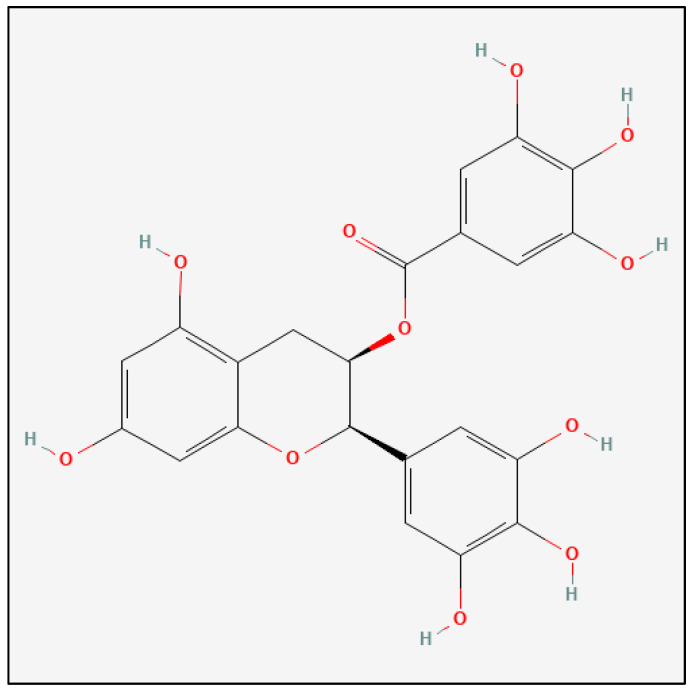
Chemical structure of epigallocatechin-3-gallate (EGCG) [16].

EGCG (C22H18O11) has the chemical structure [(2R,3R)-5,7-dihydroxy-2-(3,4,5-trihydroxyphenyl)-3,4-dihydro-2H-chromen-3-yl] 3,4,5-trihydroxybenzoate, and it is a phenolic antioxidant with a molecular weight of 458.375 g/moL (Figure 1) [16]. EGCG has demonstrated a range of pharmacological activities that may benefit MS patients, including modulation of immune cell function, inhibition of inflammatory cytokine production, and protection against oxidative stress and neuronal damage, suggesting that EGCG could potentially ameliorate the pathophysiological processes in MS [17]. Other studies report that EGCG disrupts the formation of neurotoxic structures in Alzheimer’s disease, reduces oxidative damage via key signaling pathways, and inhibits replication of various neurotropic viruses, demonstrating its multifunctional role in neurological health [18].

Therefore, the hypothesis of the current study is that EGCG supplementation, with or without standard care, will result in significant improvement in clinical outcomes and biomarkers of disease activity compared to standard care. The primary objective of this systematic review is to evaluate the efficacy and safety of EGCG as a dietary supplement in patients with multiple sclerosis. Specifically, the review aims to determine the impact of EGCG on disease progression, symptom management, and overall quality of life in MS patients. This systematic investigation will contribute to a better understanding of the role of dietary supplements in managing MS and guide future research and clinical practice.

## 2. Materials and Methods

### 2.1. Eligibility Criteria

This systematic review considered studies for the final analysis based on the following inclusion criteria: (1) Study population: Studies that involved patients diagnosed with multiple sclerosis across all age groups and both genders were included, irrespective of the stage or subtype of MS (e.g., relapsing-remitting, primary progressive). (2) Focus on dietary supplementation: Research that specifically examined the use of epigallocatechin-3-gallate as a dietary supplement was selected. These studies needed to explicitly address the impact of EGCG on clinical outcomes such as disease progression, relapse rate, symptom management, and quality of life. (3) Types of studies: Included studies encompassed only clinical trials with a registration code, on the efficacy and safety of EGCG in MS. (4) Outcome measures: The primary outcome for this review was MS-related disability, measured using the EDSS. Secondary outcomes included biomarkers of inflammation, oxidative stress, and neurological function. Despite observing some symptomatic benefits, the studies primarily showed no significant change in MS-related disability, confirming the overall negative impact on disease progression. (5) Language: This review was limited to peer-reviewed articles published in English, to ensure the feasibility of us conducting a thorough review and analysis. The main study outcome, EDSS, is a standardized measure used to assess and monitor the level of disability in individuals with multiple sclerosis. The scale ranges from 0, indicating no disability, to 10, representing death due to MS. It evaluates multiple functional systems to determine scores, including mobility, where scores of 4.0 to 7.5 specifically reflect the patient’s ability to walk.

The exclusion criteria comprised the following: (1) Non-human studies: Research not involving human participants, such as in vitro or animal model studies on MS, were excluded to focus solely on human patient experiences and outcomes. (2) Broad dietary focus: Studies that did not specifically examine the impact of EGCG supplementation or those that did not differentiate the effects of EGCG from other treatments or supplements were excluded. (3) Lack of specific outcomes: Studies that did not provide clear, quantifiable outcomes related to the efficacy or safety of EGCG in MS, or lacked sufficient detail for a comprehensive analysis, were excluded. (4) Gray literature: To maintain the credibility and reliability of the data included in the review, gray literature, including non-peer-reviewed articles, preprints, conference proceedings, general reviews, commentaries, and editorials, were excluded. (5) Types of studies: Observational studies, clinical trials, cohort studies, case–control studies, and cross-sectional studies were excluded.

### 2.2. Information Sources

To conduct a comprehensive and systematic review of the literature on the efficacy and safety of epigallocatechin-3-gallate as a dietary supplement in patients with multiple sclerosis, this study implemented an exhaustive search strategy across several key electronic databases. The databases searched included PubMed, Scopus, and the Web of Science library. The literature search was targeted to include publications up to 23 February 2024, ensuring the inclusion of the most recent and relevant studies on the topic. The primary objective of the search strategy was to collate studies that evaluated the clinical outcomes, immune modulation, and neuroprotective effects of EGCG supplementation in MS, focusing on randomized controlled trials, observational studies, and clinical trials. This approach was designed to provide a broad and in-depth understanding of the potential role and benefits of EGCG in managing multiple sclerosis, enhancing the reliability and validity of the review findings.

### 2.3. Search Strategy

The search strategy for this systematic review was carefully developed to encompass a wide range of literature on the use of epigallocatechin-3-gallate in treating multiple sclerosis. The keywords were selected to capture the broad aspects of dietary supplementation, neuroprotection, immune system modulation, and specific clinical outcomes related to MS. Key search terms included “epigallocatechin-3-gallate”, “EGCG”, “multiple sclerosis”, “MS”, “dietary supplements”, “neuroprotection”, “immune modulation”, “antioxidant therapy”, “inflammatory cytokines”, “neuroinflammation”, “disease progression”, “relapse management”, “symptom reduction”, “quality of life improvements”, “oxidative stress markers”, “neurological function”, “patient-reported outcomes”, “biomarkers”, and “therapeutic efficacy”.

To ensure a comprehensive and precise literature retrieval, Boolean operators (AND, OR, NOT) were strategically used to refine the search. The detailed search string was constructed as follows: (((“epigallocatechin-3-gallate” OR “EGCG”) AND (“multiple sclerosis” OR “MS”)) AND (“dietary supplements” OR “nutritional supplementation” OR “neuroprotection” OR “immune modulation”) AND (“clinical outcomes” OR “disease progression” OR “relapse management” OR “symptom reduction” OR “quality of life”) AND (“antioxidant therapy” OR “neuroinflammation” OR “oxidative stress markers” OR “neurological function” OR “patient-reported outcomes” OR “biomarkers” OR “therapeutic efficacy”)). This approach aimed to filter studies that addressed the various dimensions of EGCG’s potential impact on MS, focusing on both the mechanistic insights and clinical benefits, thereby ensuring the inclusion of relevant and high-quality studies in the review.

### 2.4. Data Collection and Selection Process

Following the Preferred Reporting Items for Systematic Reviews and Meta-Analyses (PRISMA) guidelines [19], the selection process for this review was structured and transparent to guarantee the reproducibility and reliability of our findings. Initially, all retrieved articles were independently screened by two reviewers to ascertain their eligibility based on the predefined inclusion and exclusion criteria. The initial screening involved assessing titles and abstracts to filter out irrelevant studies.

Any discrepancies between the reviewers during this phase were resolved through detailed discussion, and if agreement could not be reached, a third reviewer was consulted to make a final decision. Full texts of potentially relevant studies were then retrieved and independently assessed by the same reviewers to confirm their inclusion in the final analysis. We employed reference management and systematic review software to manage the citations and track the screening process, which improved efficiency and minimized the risk of manual errors.

The review protocol, including the detailed selection methodology, was registered and is publicly accessible on the Open Science Framework (OSF) with the registration code osf.io/zqjb6. This registration enhances the transparency and accessibility of our research methodology and findings, allowing for verification and replication by other researchers in the field.

### 2.5. Data Items

The data collected from each study included the following: (1) Clinical outcomes: Measures such as the EDSS scores, annualized relapse rates, MRI lesion activities, and other neurological functional parameters were central. These outcomes provided insights into the disease progression and the neuroprotective effects of EGCG. (2) Biomarkers of disease activity: We collected data on inflammatory cytokines like interleukin-6, oxidative stress markers, and other relevant immunological and biochemical indices. This allowed for an assessment of the internal biochemical changes and immune response modifications resulting from EGCG supplementation. (3) Patient-reported outcomes: Information on quality of life, symptom management, and functional abilities, including mobility and cognitive function, was gathered. These outcomes are critical for evaluating the real-world effectiveness of EGCG in enhancing the daily lives of MS patients. (4) Safety profiles: Data on adverse events, treatment tolerability, and long-term safety were also collected to evaluate the risks associated with EGCG supplementation in MS patients. Additionally, to provide context and enhance the reliability of our findings, we gathered data on study characteristics such as the country, study year, design, and study quality, along with population characteristics, including age, gender distribution, stage of MS, and time since diagnosis. These demographic and clinical variables helped us to understand who benefits most from EGCG treatment and under what circumstances. We also noted the dosages of EGCG used and the duration of supplementation, to assess the dose–response relationship and the optimal treatment regimen.

### 2.6. Risk of Bias and Quality Assessment

For the systematic assessment of study quality and determination of risk of bias within the included studies, our review employed a dual approach, integrating both qualitative and quantitative evaluation methods. Initially, the quality of observational studies was evaluated using the Newcastle–Ottawa Scale [20], a widely recognized tool that assesses three critical dimensions: the selection of study groups, the comparability of these groups, and the ascertainment of either the exposure or outcome of interest for case–control or cohort studies. Each study is awarded stars in these categories, cumulating in a score that classifies the study quality as either low, medium, or high. To ensure the objectivity and reproducibility of our quality assessment process, each study was independently evaluated by two researchers. Discrepancies in quality assessment scores were resolved through discussion, or if necessary, consultation with a third researcher.

### 2.7. Synthesis Methods

In this systematic review, we integrated findings from selected studies on the effects of epigallocatechin-3-gallate in multiple sclerosis through both qualitative and quantitative syntheses, considering the variability in study designs and outcome measures reported. The selection of studies for synthesis strictly adhered to our predefined inclusion criteria, focusing primarily on the clinical outcomes associated with EGCG supplementation in MS, such as disease progression, symptom management, and quality of life improvements.

To prepare data for synthesis, we performed a detailed tabulation of key outcomes including immune response measures, neuroprotective effects, and patient-reported outcomes. Missing data were noted explicitly, and the potential impacts of these absences on our findings were acknowledged. Results from individual studies were summarized and presented descriptively, enabling a comparative analysis of clinical effectiveness and safety outcomes across different geographical and clinical settings.

## 3. Results

### 3.1. Study Selection and Study Characteristics

**Figure 2:**
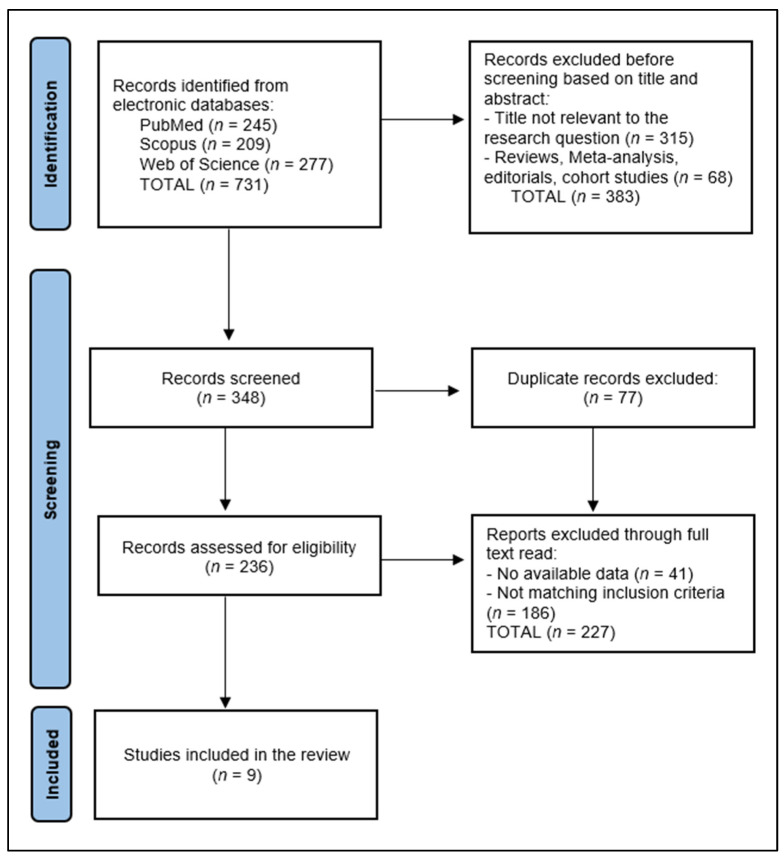
PRISMA flow diagram for systematic review of EGCG clinical trials.

A total of 731 articles were identified according to the initial search, of which 77 duplicate entries were eliminated, 383 records excluded before screening based on title and abstract, and 227 articles excluded after a full read for not matching the inclusion criteria or having no available data, as presented in Figure 2.

The systematic review examined the effects of epigallocatechin-3-gallate supplementation on multiple sclerosis management, incorporating nine clinical trials conducted between 2015 and 2023 [21,22,23,24,25,26,27,28,29]. The research predominantly took place in Europe, with trials concentrated in Germany and Spain, reflecting regional interest in this therapeutic approach. The timeline of these studies ranged from the earlier work of Mähler et al. [23] in 2015 to more recent research by de la Rubia Ortí et al. [25] and Cuerda-Ballester et al. [27] in 2023, demonstrating an ongoing interest and progression in the investigation of EGCG as a treatment option.

Regarding the quality of these studies, the majority were classified as high quality (six out of nine), indicating rigorous data collection and analysis procedures. High-quality ratings were assigned to studies by Bellmann-Strobl et al. [22], Benlloch et al. [24], Rust et al. [26], Platero et al. [28], and both studies by de la Rubia Ortí et al. [25,29] in 2021 and 2023. In contrast, studies by Platero et al. [21] in 2020 and Cuerda-Ballester et al. [27] in 2023 were rated as medium quality, indicating certain limitations in design or execution that could have affected the consistency and reliability of the findings (Table 1).

The studies included a total of 318 participants, with sample sizes ranging from 18 in Mähler et al. [23] to 62 in Bellmann-Strobl et al. [22]. The age of participants varied from as young as 18 to as old as 65, with a mean age typically around 44–45 years across most studies, suggesting that middle-aged individuals were predominantly involved in these trials. Notably, Platero et al. [21], Benlloch et al. [24], and two studies by de la Rubia Ortí et al. [25,29] reported a mean age of approximately 44.5 years.

The gender distribution across the studies often leaned towards a higher proportion of female participants, which aligns with the higher incidence of multiple sclerosis in females. This was particularly evident in studies like de la Rubia Ortí et al. [25,29] and Platero et al. [28], where females constituted over 80% of the participants.

BMI measurements were detailed in several studies, with most reporting values around the typical range for adults, thereby indicating that the average physical condition of the participants did not deviate significantly from the norm. Mähler et al. [23] provided separate post-EGCG and post-placebo BMI values, showing minimal change, which could suggest that short-term EGCG supplementation has little impact on body weight or composition.

**Figure 3:**
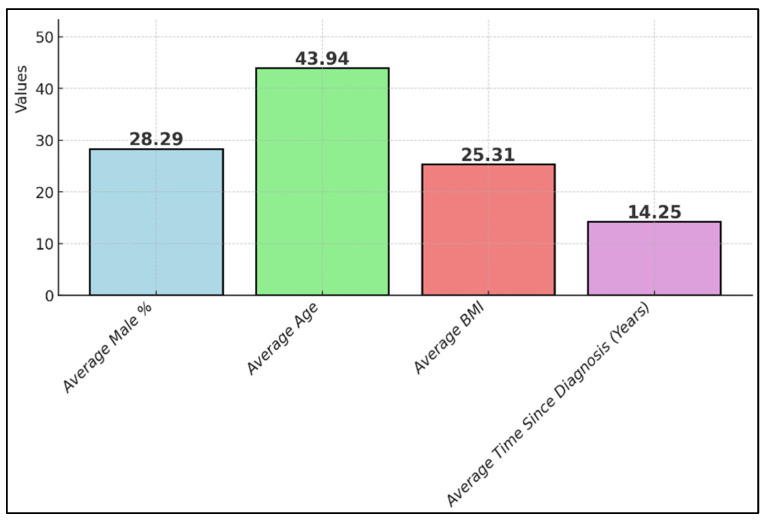
Composite summary of background characteristics of study participants.

The duration of illness before the study commencement varied widely, from a minimum of six months as reported by Benlloch et al. [24] to a median of 14.5 years in Cuerda-Ballester et al. [27]. This variation in disease duration provided a diverse base for evaluating the effects of EGCG supplementation across different stages of multiple sclerosis progression (Table 2 and Figure 3).

### 3.2. Results of Synthesis

Platero et al. [21] administered 800 mg of EGCG combined with 60 mL of coconut oil over a period of 4 months. The Expanded Disability Status Scale scores remained unchanged, suggesting no improvement in disability status. However, significant reductions in interleukin-6 and state anxiety levels were observed, indicating that the combination could have anti-inflammatory and anxiolytic benefits. Bellmann-Strobl et al. [22], utilizing a longer follow-up period of 18 months with a daily dose of 800 mg of EGCG, reported minimal change in EDSS scores and no significant differences in annualized relapse rates or MRI lesion activity compared to the placebo. This suggested that EGCG, even when used long-term, might not affect disease progression significantly when measured by these parameters.

Mähler et al. [23] observed sex-specific responses over 12 months of 600 mg EGCG daily administration, noting improved fat oxidation and exercise efficiency, particularly in men. This could indicate potential benefits of EGCG on metabolic functions in multiple sclerosis patients, which might vary by sex. Benlloch et al. [24] and Cuerda-Ballester et al. [27], both administering 800 mg of EGCG with 60 mL of coconut oil daily for 4 months, found improvements in metabolic health markers and functional abilities such as gait speed and balance. These results suggest that EGCG might contribute to enhanced physical functionality and reduced cardiovascular risk in multiple sclerosis patients. However, it is essential to understand that many studies analyzed EGCG in combination with coconut oil [21,24,25,27,28,29]. Therefore, the confounding effect should be considered when interpreting the findings.

de la Rubia Ortí et al. [25,29] noted positive effects on lipid metabolism and muscle mass, along with reductions in inflammatory markers. These changes point to potential systemic benefits of EGCG supplementation, particularly in managing symptoms or co-morbidities associated with multiple sclerosis. Rust et al. [26], with the highest dosage of 1200 mg daily over an extended period of 36 months, did not meet their primary endpoint of reducing brain atrophy, indicating that higher doses over longer periods may not necessarily translate into clinically significant neuroprotective effects. Platero et al. [28] found significant reductions in depression levels, adding to the evidence that EGCG could have various neuropsychological benefits, as can be seen in Table 3.

The combined treatment of EGCG and coconut oil appears to offer distinct benefits over EGCG alone in the management of multiple sclerosis, as evidenced by the clinical trials included in this systematic review. For instance, the study by Platero et al. [1] demonstrated that the addition of coconut oil to an 800 mg daily dose of EGCG resulted in significant reductions in interleukin-6 and state anxiety levels, suggesting that the combination may enhance the anti-inflammatory and anxiolytic effects of EGCG. Similarly, Benlloch et al. [24] and Cuerda-Ballester et al. [27] found that this combination improved metabolic health markers and functional abilities, such as gait speed and balance, potentially due to the synergistic effects of the two components.

In contrast, studies administering EGCG alone, such as that by Bellmann-Strobl et al. [22], did not demonstrate significant changes in disease progression metrics like EDSS scores or MRI lesion activity over 18 months. This lack of significant improvement with EGCG alone might indicate that while EGCG has potential benefits, its efficacy could be enhanced by the addition of coconut oil, which might influence metabolic and inflammatory pathways more effectively.

These findings suggest a compelling case for further research into the combination of EGCG and coconut oil as a therapeutic approach, potentially providing a more comprehensive management strategy for multiple sclerosis that addresses both neurodegenerative and systemic aspects of the disease.

## 4. Discussion

### 4.1. Summary of Evidence

This systematic review exploring the efficacy of epigallocatechin-3-gallate supplementation in managing multiple sclerosis provides a nuanced understanding of its potential therapeutic effects. Although the primary endpoint of reducing disease progression, as measured by the EDSS, was not significantly impacted in most studies, noteworthy benefits in other clinical and biomarker outcomes were observed. For instance, EGCG, particularly when combined with coconut oil, consistently showed improvements in metabolic health markers and functional abilities like gait speed and balance. These findings suggest a supportive, albeit indirect, role of EGCG in enhancing physical functionality and potentially ameliorating cardiovascular risks in MS patients. Furthermore, observed reductions in inflammatory markers and depression levels indicate that EGCG could also confer anti-inflammatory and neuropsychological benefits, which are crucial for managing the broader symptomatic landscape of MS. These aspects underscore the potential utility of EGCG as part of a comprehensive management strategy for MS, focusing on symptom relief and overall quality of life improvement.

The inclusion of coconut oil alongside EGCG in several studies under review warrants attention, particularly regarding its impact on triglyceride levels. Some studies reported a decline in TG levels, which may not solely be attributed to EGCG but rather to the effects of coconut oil, known for its lipid-modifying properties, although different studies showed both an increase and a decrease in lipid levels [30,31]. This confounding factor highlights the need for careful interpretation of the results, as it complicates the assessment of EGCG’s direct effects on MS-related biomarkers and overall health outcomes. Future research should consider isolating the effects of EGCG from those of coconut oil to delineate their respective impacts more clearly.

Strategies known to enhance the bioavailability of EGCG include nanostructure-based drug delivery systems and molecular modifications [32]. Our findings indicate that encapsulating tea catechins in protein-based, carbohydrate-based, and lipid-based nanoparticles significantly improves their stability, sustainable release, and cell membrane permeation, leading to increased bioavailability. Furthermore, we investigated the potential of coconut oil as a co-administered lipid-based agent. Our results suggest that coconut oil may enhance the bioavailability and stability of catechins.

The demographic and clinical characteristics of participants in studies evaluating EGCG’s efficacy in treating multiple sclerosis are reflective of a predominantly female sample, which aligns with the higher incidence of MS in women. Participants’ ages generally range from mid-30s to early 50s, with BMIs typically in the normal to slightly overweight range. Studies conducted in Spain and Germany between 2015 and 2023, predominantly randomized trials, feature a diverse MS patient base with disease durations varying from several months to over two decades. The study quality was consistently rated high, except for two trials, which received a medium quality rating. This comprehensive representation across various demographics and disease stages highlights the potential utility and broad applicability of EGCG in different MS patient profiles.

The findings also highlighted sex-specific responses to EGCG, particularly in metabolic functions. Studies like that of Mähler et al. [23] demonstrated increased fat oxidation and exercise efficiency in male participants over a 12-month period. This sex-specific variance underscores the need for personalized treatment approaches in MS, considering the metabolic and physiological differences among patients. Additionally, the consistent reduction in markers such as interleukin-6 and improvements in lipid metabolism and muscle mass across several studies support the systemic benefits of EGCG, potentially aiding in the broader management of MS symptoms and co-morbidities.

Statistically, the consistent inclusion of middle-aged, predominantly female participants across trials with varying durations of disease offers a comprehensive dataset for analyzing the therapeutic effects of EGCG. The variety in disease duration and minimal variance in BMI before and after trials might indicate that while EGCG’s effects on physical dimensions are limited, its potential benefits could be more pronounced in other clinical aspects of multiple sclerosis management, such as symptom severity and progression, which were not detailed in the table but could be inferred from the long-term trial engagement of participants with the condition.

Other studies such as that by Cai et al. [32] demonstrate that EGCG can modulate immune responses directly by altering macrophage subtypes within the central nervous system, significantly reducing EAE severity and macrophage inflammation. This particularly emphasizes the shift from M1 (pro-inflammatory) to M2 (anti-inflammatory) macrophages, suggesting an immunomodulatory mechanism mediated by the inhibition of NF-κB signaling and macrophage metabolic pathways involved in glycolysis. Conversely, the study by Herges et al. [33] explores a combination therapy approach, where EGCG paired with glatiramer acetate (GA), a known immunomodulator, provides not only enhanced neuroprotective effects but also demonstrates neuro-regenerative properties. Their results show significant delays in disease onset, a reduction in clinical severity post symptom onset, and reductions in inflammatory infiltrates, highlighting synergistic effects that extend beyond simple immunomodulation to include direct neuroprotection and regeneration of neuronal structures. Another study [18] described that EGCG disrupts amyloid-beta aggregation in Alzheimer’s disease, enhances protective α-helix structures, and modulates critical neuronal pathways such as GSK3-β and PI3K/Akt to reduce oxidative stress. Additionally, EGCG inhibits the entry and replication of neurotropic viruses, supporting its role in neuroprotection and anti-viral defenses.

Moreover, β-hydroxybutyric acid (BHB) is particularly relevant as it reflects metabolic changes that could benefit MS management. BHB not only serves as an alternative energy source during periods of low glucose availability but also exhibits neuroprotective and anti-inflammatory properties. These effects are crucial for MS, where inflammation plays a significant role in disease progression [34]. The increasing BHB levels observed in a previous study suggest that EGCG supplementation may enhance ketone body production, potentially providing additional neuroprotection and metabolic benefits to MS patients.

Other research provides insights into the therapeutic effects of epigallocatechin-3-gallate across different neurological disorders, focusing on molecular mechanisms in animal models. Semnani et al. [35] reported significant increases in the markers of remyelination, proteolipid protein, and oligodendrocyte transcription factor 1 in a multiple sclerosis model, indicating robust remyelination after 2 and 4 weeks of EGCG treatment compared to controls. In contrast, Bimonte et al. [36] explored EGCG’s potential in neuropathic pain (NP), noting its anti-inflammatory and antioxidant properties, which contribute to pain reduction in bone cancer pain models. This contrast highlights EGCG’s broad applicability, from promoting neural repair in demyelinating diseases like MS to alleviating pain by modulating inflammatory responses in NP, showcasing its diverse therapeutic mechanisms in neurological pathologies.

On the study of different biological markers and outcomes in MS, Klumbies et al. [37] analyzed the impact of EGCG on retinal thickness in progressive MS patients, revealing no significant differences in changes in the peripapillary retinal nerve fiber layer (pRNFL), ganglion cell/inner plexiform layer, and inner nuclear layer thicknesses between EGCG-treated and placebo groups over 2 years. Specifically, mean changes in pRNFL were −0.83 μm in the EGCG group versus −0.64 μm in the placebo group, with *p*-values indicating no statistical significance. Conversely, Afshar et al. [38] evaluated the influence of EGCG on gene expression in peripheral blood mononuclear cells, finding a significant decrease in RORC2 gene expression, which suggests an immunomodulatory effect, but no change in HIF-1α levels. These contrasting findings highlight the specificity of EGCG’s effects depending on the target and measurement context—showing limited structural neuroprotection in retinal tissues but effective immunomodulation at the gene expression level in immune cells.

Mische and Mowry critically evaluated various diets and nutritional supplements, finding inconclusive results from mostly observational studies, with no strong evidence supporting a direct benefit of any specific diet on MS prognosis [39]. They highlight a potential benefit of biotin in progressive MS based on pilot data but emphasize the lack of significant impacts from the best-designed randomized controlled trials, particularly concerning polyunsaturated fatty acid supplementation. In contrast, Katz Sand [40] investigated the broader potential impacts of dietary factors on MS incidence and progression, suggesting that components like fatty acids and dietary patterns such as the Mediterranean diet might influence disease outcomes. Katz Sand’s review points to preliminary evidence suggesting potential benefits and calls for larger clinical trials to confirm these findings. Both reviews underscore the need for higher-quality trials to clarify the role of diet in MS, but Katz Sand offers a slightly more optimistic view on the potential of dietary interventions as disease modifiers, backed by emerging evidence from various study designs.

Other reviews offer contrasting perspectives primarily in terms of evidence strength and specific dietary recommendations. Evans et al. [41] focus on the role of vitamins and dietary supplements in MS, noting that while many patients are interested in these as potential treatments, the only supplement with substantial evidence supporting its use is vitamin D. They highlight ongoing large randomized clinical trials for biotin and vitamin D, and smaller trials for other supplements like lipoic acid and probiotics, but indicate that most human trials thus far have been limited by small sizes and non-blinded methodologies. Conversely, Mandato et al. [42] discuss broader dietary and nutritional issues, including the impact of overall diet quality and specific nutrients like vitamin D and polyunsaturated fatty acids on MS, particularly emphasizing their roles in pediatric MS. They advocate for balanced diets rich in fruits, vegetables, whole grains, and lean proteins, noting these diets’ benefits due to their anti-inflammatory properties and positive effects on gut microbiota, which are crucial for maintaining intestinal and brain barrier integrity.

Nevertheless, as of the present day, EGCG, as a major component of green tea, is not officially listed as a dietary supplement for the adjunctive treatment of MS in authoritative clinical guidelines or recommendations. Clinical use of supplements like EGCG for MS typically awaits robust evidence from large-scale clinical trials and endorsement from regulatory bodies or professional medical associations.

To enhance data extraction in clinical trials, adaptive trial designs can be employed for real-time protocol optimizations based on interim results. Incorporating digital health tools, such as wearable technology, allows for continuous monitoring of patients’ bodily functions, providing a richer dataset. Additionally, using advanced statistical techniques like machine learning can help analyze complex datasets, uncovering new patterns that traditional methods might overlook.

### 4.2. Limitations

This article acknowledges several limitations to the review conducted. Firstly, the variability in study designs and outcome measures across the included trials introduced heterogeneity, which complicated the synthesis of data and might have masked more subtle effects of EGCG supplementation. Furthermore, the reliance on self-reported outcomes in some studies could have introduced bias and affected the reliability of the results. Moreover, the studies’ focus on short- to medium-term outcomes left the long-term efficacy and safety of EGCG supplementation largely unexplored, which is crucial for chronic conditions like MS. So far, the minimal changes in EDSS scores and MRI lesion activity, as noted in studies with longer follow-up periods, raise questions about the direct neuroprotective effects of EGCG. While secondary outcomes related to quality of life and symptom management show promise, the lack of a significant impact on the primary disease metrics cannot be overlooked.

Moreover, the variable dosages and combination with coconut oil in several studies hint at potential synergistic effects that merit further investigation. Other significant limitations in the research on EGCG for multiple sclerosis include its low bioavailability and poor penetration of the blood–brain barrier. Moreover, many studies exhibited methodological weaknesses, primarily using inadequate intra-group analyses without comparing changes between control and intervention groups. Additionally, the concentration of research efforts by a single group further limits the generalizability of the findings, casting doubt on the efficacy of EGCG in treating MS.

## 5. Conclusions

Recent randomized trials from Spain and Germany (2015–2023) on EGCG’s role in MS show its potential across a wide demographic, primarily involving women, and consistently indicate a high study quality. However, EGCG trials have demonstrated mixed outcomes in multiple sclerosis management. Key findings include significant reductions in IL-6 levels and state anxiety, improvements in metabolic health markers like fat oxidation and muscle mass, and positive shifts in lipid metabolism. However, several studies noted no significant change in disability progression as measured by EDSS or MRI outcomes, suggesting that while EGCG may improve certain metabolic and psychological parameters, its impact on disease progression remains inconclusive. While EGCG supplementation shows promise in improving symptomatic management and quality of life for MS patients, its impact on primary disease progression metrics like EDSS scores and MRI lesions remains minimal. Moreover, this systematic review has revealed methodological limitations in the studies analyzed, including short follow-up periods and variable dosing, which may have obscured the true long-term efficacy of EGCG. Future research should prioritize longer-term studies with standardized dosing protocols to better assess EGCG’s potential as a disease-modifying treatment. Additionally, incorporating novel methodologies such as adaptive trial designs could enhance the extraction of significant data, potentially revealing more about the compound’s therapeutic capabilities.

## Figures and Tables

**Table 1:** Detailed characteristics of studies evaluating EGCG in multiple sclerosis.

Study and Author	Country	Study Year	Study Design	Study Quality
1 [21] Platero et al.	Spain	2020	Randomized trial	Medium
2 [22] Bellmann-Strobl et al.	Germany	2021	Randomized trial	High
3 [23] Mähler et al.	Germany	2015	Randomized trial	High
4 [24] Benlloch et al.	Spain	2020	Randomized trial	High
5 [25] de la Rubia Ortí et al.	Spain	2023	Randomized trial	High
6 [26] Rust et al.	Germany	2021	Randomized trial	High
7 [27] Cuerda-Ballester et al.	Spain	2023	Randomized trial	Medium
8 [28] Platero et al.	Spain	2021	Randomized trial	High
9 [29] de la Rubia Ortí et al.	Spain	2021	Randomized trial	High

**Table 2:** Demographic and clinical characteristics of participants across studies.

Study Number	Sample Size (Intervention Group)	Mean Age/Age Range	Gender Distribution	Weight/BMI	Time Since Diagnosis
1 [21] Platero et al.	27	45 years	18.5% male, 81.5% female	Pre-test BMI 23.43 kg/m^2^, post-test BMI 23.49 kg/m^2^	Range 9–35 years
2 [22] Bellmann-Strobl et al.	62	39 years	67% female, 33% male	NR	Median 6.1 years
3 [23] Mähler et al.	18 (8 men and 10 women)	40 years for men, 45 years for women	44.4% male, 55.6% female	Men 24.7 kg/m^2^, women 25.9 kg/m^2^ post-EGCG; men 24.0 kg/m^2^, women 25.8 kg/m^2^ post-placebo	89 months (7–192) for men, 60 months (25–208) for women
4 [24] Benlloch et al.	51	44.5 years	58.3% female, 41.7% male	Mean BMI: 25.92 kg/m^2^	At least 6 months
5 [25] de la Rubia Ortí et al.	25	44 years	20% male, 80% female	25.9 kg/m^2^	Mean 11.7 years
6 [26] Rust et al.	30	18–65 years	NR	NR	NR
7 [27] Cuerda-Ballester et al.	51	50 years	31.7% male, 58.3% female	18.9% fat mass	Median 14.5 years
8 [28] Platero et al.	27	44.5 years	18.5% male, 81.5% female	19.4% fat mass	Mean 11.8 years
9 [29] de la Rubia Ortí et al.	27	44.5 years	18.5% male, 81.5% female	25.97 kg/m^2^	Mean 12 years

NR—not reported; BMI—Body Mass Index.

**Table 3:** Summary of clinical outcomes in EGCG trials.

Study Number	Measurement/Dose/Administration	Follow-Up	Multiple Sclerosis Disease Features (Activity/Disability)	Therapeutic Effects	Interpretation
1 [21] Platero et al.	800 mg of EGCG and 60 mL of coconut oil	4 months	EDSS pre-test: 3.00 EDSS Post-test: 3.00 (indicating no significant change in disability status)	IL-6: pre-test (pg/mL): 2.18, post-test (pg/mL): 0.84 (significant reduction) State anxiety (STAI): pre-test: 23.00, post-test: 17.00 (significant reduction)	Both EGCG and coconut oil contribute to reducing state anxiety and IL-6 levels, with a slight improvement in functional capacity
2 [22] Bellmann-Strobl et al.	800 mg of EGCG daily	18 months	EDSS score at baseline: median 2.0, range 0–6.0 EDSS score at 18 months: median 2.2, change from baseline 0.14	Annualized relapse rate: EGCG group: 0.47, placebo group: 0.50 MRI lesion activity: proportion of patients without new T2w lesions at 18 months: EGCG group: 29% (18 of 62 patients), placebo group: 25% (15 of 60 patients) (no significant difference) Number of new T2w lesions: EGCG group: mean 3.1, placebo group: mean 1.9 (no significant difference)	EGCG, added to glatiramer acetate, did not demonstrate superiority over placebo in reducing MRI and clinical disease activity over 18 months. It was safe to use at the tested dosage
3 [23] Mähler et al.	600 mg of EGCG daily	12 months	EDSS score: ≤4.5 for all participants	Therapeutic effects: fat oxidation (FAOx) at rest (postprandial, g/4 h): placebo: men 9.2 ± 4.9, women 7.8 ± 4.2; EGCG: men 12.9 ± 5.7, women 6.2 ± 3.6 Energy expenditure efficiency during exercise (%): placebo: men 21 ± 3, women 20 ± 3; EGCG: men 27 ± 6, women 25 ± 7	EGCG administration led to a sex-specific response in energy metabolism at rest and during exercise. In men, EGCG increased fat oxidation and exercise efficiency more than in women. The therapeutic effects suggest that EGCG may improve metabolic function in MS patients, particularly in men
4 [24] Benlloch et al.	800 mg of EGCG and 60 mL of coconut oil daily	4 months	EDSS pre-test: 3.80 ± 2.00, EDSS post-test: 3.37 ± 2.03	WHR decreased from 0.95 to 0.87, WHtR decreased from 0.60 to 0.55, fat mass decreased from 19.01% to 17.74%, muscle mass increased from 38.01% to 41.10%, albumin increased from 4.55 g/dL to 4.83 g/dL, BHB increased from 0.04 mMol/L to 0.10 mMol/L, PON1 increased from 2.49 UI/L to 2.97 UI/L	EGCG combined with coconut oil significantly altered cardiac risk markers, improving metabolic health in MS patients, potentially lowering cardiovascular risk
5 [25] de la Rubia Ortí et al.	800 mg of EGCG and 60 mL of coconut oil	4 months	NR	Triglycerides: control: pre: 112.8, post: 142 mg/dL; intervention: pre: 103.6, post: 88.7 mg/dL Total cholesterol (TC): control: pre: 210.8, post: 236.4 mg/dL; intervention: pre: 220.9, post: 232.5 mg/dL HDL cholesterol: control: pre: 76.56, post: 86 mg/dL; intervention: 84.81 mg/dL	EGCG and coconut oil can positively influence lipid metabolism, particularly by reducing triglyceride levels, which correlates with improvements in functional disability in MS patients
6 [26] Rust et al.	1200 mg of EGCG daily	36 months with an optional 12-month extension	EDSS at screening was 3–8.37% in the EGCG group and 39% in the placebo group (with primary progressive disease)	Study did not meet its primary endpoint as the rate of decrease in brain parenchymal fraction over 36 months was 0.0092 in the EGCG group and −0.0078 in the placebo group. Annualized atrophy rates (AARs) were 0.31% for the EGCG group and 0.26% for the placebo group	Primary endpoint of reducing brain atrophy not met; no significant changes in MRI and clinical end points
7 [27] Cuerda-Ballester et al.	800 mg of EGCG and 60 mL of coconut oil daily	4 months	EDSS significant improvement from 3.7	Berg balance scale score increasing from an initial average of 49 ± 9.6 to 52 ± 6.9 after treatment; significant improvement in the intervention group, where the 10 m walk test (10 MWT) times improved from 1.56 ± 0.58 m/s to 1.73 ± 0.61 m/s; 2 WMT distance increased from 113 ± 38 m to 136 ± 41 m	EGCG and coconut oil improve gait speed and balance, contributing to enhanced functionality in MS patients
8 [28] Platero et al.	800 mg of EGCG and 60 mL of coconut oil daily	4 months	NR	Depression (BDI-II scale) decreased from a median of 12.0 to 8.0	Significant reduction in depression levels and abdominal fat; increase in albumin levels in the intervention group
9 [29] de la Rubia Ortí et al.	800 mg of EGCG and 60 mL of coconut oil daily	4 months	NR	BHB levels increased from 0.05 to 0.10 mMol/L, muscle mass increased significantly, IL-6 levels decreased	Intervention showed improvements in muscle mass and reductions in inflammation markers, indicating positive therapeutic effects

NR—not reported; IL—interleukin; 2 MWT—Two-Minute Walking Test.
